# Immunomodulation and Antioxidant Activities as Possible Trypanocidal and Cardioprotective Mechanisms of Major Terpenes from *Lippia alba* Essential Oils in an Experimental Model of Chronic Chagas Disease

**DOI:** 10.3390/antiox10111851

**Published:** 2021-11-22

**Authors:** Denerieth Ximena Espinel-Mesa, Clara Isabel González Rugeles, Julio César Mantilla Hernández, Elena E. Stashenko, Carlos Andrés Villegas-Lanau, John Jaime Quimbaya Ramírez, Liliana Torcoroma García Sánchez

**Affiliations:** 1 Infectious Diseases Postgraduate Program, Instituto de Investigación Masira, Universidad de Santander, Bucaramanga 680006, Santander, Colombia; buc19861011@mail.udes.edu.co (D.X.E.-M.); jo.quimbaya@mail.udes.edu.co (J.J.Q.R.); 2 Immunology and Molecular Epidemiology Group, School of Microbiology, Universidad Industrial de Santander, Bucaramanga 680002, Santander, Colombia; cig@uis.edu.co; 3 Pathology Department, School of Medicine, Universidad Industrial de Santander, Bucaramanga 680002, Santander, Colombia; jumantil@uis.edu.co; 4 National Research Center for the Agroindustrialization of Tropical Aromatic and Medicinal Plant Species—CENIVAM, Universidad Industrial de Santander, Bucaramanga 680002, Santander, Colombia; elena@tucan.uis.edu.co; 5 Neurosciences Group of Antioquia, Brain Bank, Universidad de Antioquia, Medellín 050010, Antioquia, Colombia; andres.villegas@udea.edu.co

**Keywords:** chronic Chagas disease, *Trypanosoma cruzi*, antioxidant, immunomodulation, immunohistochemistry, essential oils, *Lippia alba*, limonene, caryophyllene oxide

## Abstract

In the late phase of *Trypanosoma cruzi* infection, parasite persistence and an exaggerated immune response accompanied by oxidative stress play a crucial role in the genesis of Chronic Chagasic Cardiomyopathy (CCC). Current treatments (Benznidazole (BNZ) and Nifurtimox) can effect only the elimination of the parasite, but are ineffective for late stage treatment and for preventing heart damage and disease progression. In vivo trypanocidal and cardioprotective activity has been reported for *Lippia alba* essential oils (EOs), ascribed to their two major terpenes, limonene and caryophyllene oxide. To investigate the role of antioxidant and immunomodulatory mechanisms behind these properties, chronic-*T. cruzi*-infected rats were treated with oral synergistic mixtures of the aforementioned EOs. For this purpose, the EOs were optimized through limonene-enrichment fractioning and by the addition of exogenous caryophyllene oxide (LIMOX) and used alone or in combined therapy with subtherapeutic doses of BNZ (LIMOXBNZ). Clinical, toxicity, inflammatory, oxidative, and parasitological (qPCR) parameters were assessed in cardiac tissue. These therapies demonstrated meaningful antioxidant and immunomodulatory activity on markers involved in CCC pathogenesis (IFN-γ, TNF-α, IL-4, IL-10, and iNOS), which could explain their significant trypanocidal properties and their noteworthy role in preventing, and even reversing, the progression of cardiac damage in chronic Chagas disease.

## 1. Introduction

Chagas disease is a parasitosis caused by *Trypanosoma cruzi* that affects approximately 10 million people worldwide, of whom around 30% will eventually develop organomegaly of the digestive tract or heart [1]. Dilated cardiomyopathy, or Chagas heart disease (CHD), is the most relevant manifestation of this infection during its chronic phase [1], making it the most prevalent cardiac infection in the world, and causing a significant public health problem in Latin American countries where it is endemic [2]. Because of the silent course of this parasitosis and the late appearance of its symptoms (usually 8–10 years after acquiring the parasite, and without any other pathognomonic signs), CHD can be confused with other etiologies, delaying timely diagnosis [3]. These factors combined have contributed to CHD being presently considered a neglected and high-cost disease, with an average cost of care for chronic cases estimated at USD $44,955 per person [4].

Progression of the disease is characterized by the persistence of the parasite in smooth muscle, and is associated with an exacerbated immune response and a permanent oxidative environment. Research indicates that these conditions together contribute to tissue, neurological, and microvasculature injury; with a consequent deterioration of contractile capacity and dilation of muscle tissue; which in turn can culminate in an eventual loss of the organ’s physiological function and even result in death [5]. Current treatments available for therapeutic intervention against this infection are based on the use of two nitro-heterocyclic compounds: nitrofuran Nifurtimox (NFX) and nitroimidazole Benznidazole (BNZ). These compounds, well-established for more than 50 years as the conventional therapy against *T. cruzi* infection, have been found to exhibit limited trypanocidal activity (between 50–80% in the acute phase, and 8–20% in the chronic) [6], with high toxicity due to non-selective oxidative damage.

In general terms, patients assigned therapies based on these drugs demonstrate low treatment adherence due to the presence of multiple adverse side effects (mainly severe anorexia, digestive intolerance, and neurological disorders), as well as long treatment times; all characteristics of therapeutic regimens with high rates of treatment abandonment [7]. In response, regulatory entities such as the US Food and Drug Administration (FDA) have not approved the use of NFX in human therapy, thus limiting the available prophylactic and therapeutic options to BNZ, alone, for all clinical phases of Chagas disease.

Recent studies have reported interesting in vitro trypanocidal, antioxidant, and immunomodulatory activity for fractions derived from *Lippia alba* (mill.) N.E. Brown essential oils (EOs) rich in terpenes such as limonene, citral, and caryophyllene oxide [8,9]. In addition, trypanocidal and cardioprotective qualities have also been ascribed to these oils in animals with chronic *T. cruzi* infection [10]. The aim of the present work is to assess, in an animal model of chronic Chagas disease, potential immunomodulatory and antioxidant properties as possible mechanisms for the trypanocidal and cardioprotective activity observed for these compounds. In order to accomplish this, synergistic mixtures were generated by combining a limonene-rich fraction of *L. alba* EOs with added exogenous caryophyllene oxide (LIMOX); or by the interaction of this LIMOX with subtherapeutic doses of BNZ (LIMOXBNZ). These experimental therapies were used in a daily, oral scheme of 31 doses on a murine model (Wistar rats) infected with chronic *T. cruzi*, in which CCC had been induced.

The effect of the treatments was determined through evaluation of the clinical progression of heart disease (biochemical and morphological parameters), the trypanocidal efficacy (qPCR), and by immunohistochemical analysis of the cytokine profiles relevant to the immune response against the parasite (TNF-α, IFN-γ, IL-10, and IL-4). Likewise, a marker of oxidative stress (iNOS) was also measured. The phytotherapeutics studied showed promising data for a therapy that could be used as an adjuvant to current treatments (BNZ). Such a novel therapy would be based on standardized production technologies, with Good Agricultural Practices and environmentally sustainable EOs extracted from *L. alba*, a wild shrub from the Colombian Andean Region.

## 2. Materials and Methods

### 2.1. Plant Material

Plants belonging to the specie *L. alba*, carvone chemotype, were harvested at the National Research Center for the Agroindustrialization of Tropical Aromatic and Medicinal Plant Species (CENIVAM) (Universidad Industrial de Santander (UIS)), located in Bucaramanga, Colombia, at an altitude of 960 m above sea level. For the cultivation of plant specimens, environmental conditions and specific collection times previously standardized [8] were taken into account. A plant collection permit was obtained from the Colombian Ministry of the Environment and Sustainable Development under Resolution 1761 of November 2019. The *L. alba* identification voucher was deposited at the Colombian National Herbarium (Universidad Nacional de Colombia) under Herbarium Code COL480750.

### 2.2. Extraction of Essential Oils and Their Fractions

Essential oils were obtained via steam distillation in a 0.4 m^3^ stainless steel column using fresh and mature *L. alba* leaves, and were separated by decantation. Subsequently, they were dried with anhydrous sodium sulfate and stored at 4 °C in amber flasks. The EOs were fractionated by reduced pressure distillation in a B/R Instruments (Easton, MD, USA) 800 high-efficiency microdistillation device, and enriched in the bioactive terpene (limonene). The F1 fraction rich in limonene was collected at a temperature of 67 °C and pressure of 12 Torr during distillation of the carvone-chemotype EOs, then stored in amber glass at 4 °C for later analysis.

### 2.3. GC-MS Analysis

For chromatographic and spectrometric characterization, a GC7890 Gas Chromatograph (Agilent Technologies, AT, Palo Alto, CA, USA) was used, which has an MSD 5975C mass selective detector (AT, Palo Alto, CA, USA); an electron impact ionizer (EI, 70 eV) with an aras/splitless injector (1:30 aras radius); and an MS-ChemStation G1701-DA Data System, including a WILEY, NIST, and QUADLIB 2007 spectral library. The columns used were DB-5MS fused silica capillary (J&W Scientific, Folsom, CA, USA) and DB-WAX (J&W Scientific, Folsom, CA, USA). The data obtained were reconstructed by automatic scanning, and the relative concentration of each compound was obtained by means of an AT 7890 Gas Chromatograph coupled to a flame detection system (FID, 250 °C). Identification was based upon chromatographic criteria (most importantly retention time and linear retention indices and the use of standard compounds) and spectrometric criteria (interpretation of the mass spectrum compared with standard compounds and databases) [11].

### 2.4. Animal Model

The animal model chosen was 30 male Wistar rats (*Rattus novergicus*) aged 38 ± 2 days old and with a mean weight of 65 ± 10 g, provided by the Universidad Industrial de Santander (UIS) Health Faculty’s Bioterium in Bucaramanga, Colombia. A period of eight days of acclimatization to their new environment in the animal testing laboratory at the University of Santander (UDES) was required before their usage in research. In the lab, they were housed with two animals per cage (of dimensions 24 × 37 × 24 cm), in an individually ventilated (IVC) Easy Flow cage system, with a bed of patula pine shavings, food (commercial rodent diet, Lab-Diet^®^) and sterilized water ad libitum. Variables such as humidity, temperature (21 ± 22 °C), ammonia and CO_2_ concentrations, and standardized dark/light cycles (lights on at 06:00 and off at 18:00) were controlled. For the experiments, animals were randomly assigned using the standard function = RAND in Microsoft Excel into six groups of six animals each. Three of these groups were controls: negative (not infected, not treated); hLIMOX-Control (not infected, treated with higher doses of LIMOX (EOs enriched in limonene and with added caryophyllene oxide)); and positive (infected, not treated). The three other groups were experimental: LIMOX (infected, treated with LIMOX); LIMOXBNZ (infected, treated with LIMOX + BNZ); and BNZ (infected, treated with BNZ). The positive control and experimental groups (LIMOX, LIMOXBNZ, and BNZ) were made up of animals infected with *T. cruzi*. The therapeutic schemes were administrated as described in Table 1 via daily, oral doses for 31 days continuously following the onset of the chronic disease phase as established through echocardiography and parasitemia absence. All experiments were carried out according to the NIH Guide for the Care and Use of Laboratory Animals to minimize animal pain and distress. The protocol used was approved by the Bioethics Committee of the Universidad Industrial de Santander and the Ethics Committee of the Universidad de Santander (Agreement Number 010-VII of 15 and 16 May 2017).

### 2.5. Experimental Animal Model of Chronic Chagas Disease

Animals from the positive control and experimental groups were infected using an inoculum prepared as described in the literature [10]. Briefly, 2.5 × 10^5^ metacyclic trypomastigotes of *T. cruzi* clone 338Cl2 (isolated from a confirmed CCC patient), characterized as Discrete Typing Unit (DTU) TcI, were obtained from TAU3AAG media, resuspended in a final volume of 100 µL of phosphate buffer saline-glucose (PBS-G), and injected into the intraperitoneal cavity [10]. After infection, the rats’ behavior and clinical parameters (weight, ocular perimeter, position of the vibrissae, physical condition, stool moisture, and parasitemia) were monitored every three days. Parasitemia was evaluated by observing parasites in peripheral blood obtained through puncture of the ventral coccygeal vein with a 27-gauge needle. The blood was collected in a microhematocrit tube and observed between a microscope slide and coverslip under a light optical microscope at one hundred microscopic fields (100× magnification objective) as described by Brener [12].

To determine the development and progression of CCC, the rats’ heart silhouettes were evaluated via Two-Dimensional Ultrasound, measuring the Maximum Length (ML) and Maximum Diameter (MD) along the long axis of the heart with a convex transducer and a DP20 (Mindray, Madrid, Spain) ultrasound machine. This analysis was performed at three specific points during the experiment: (i) one day prior to infection to establish baseline measurements; (ii) between 60 and 70 days after infection (d.a.i) to determine the onset of the chronic phase of the infection (presence of cardiomegaly) and to define the start date for therapy; and (iii) at the end of the treatments to evaluate the effect of the therapies applied. Animals in the experimental groups (LIMOX, LIMOXBNZ, and BNZ) received doses of the assigned treatments (Table 1) in a daily, oral scheme for 31 consecutive days, and at a specific hour (7:00 a.m.).

The test subjects were euthanized one day after the end of treatment (99 d.a.i) using an anesthetic mixture of ketamine at 90 mg/kg and xylazine at 7.5 mg/kg intraperitoneally, providing hypnosis, analgesia, and muscle relaxation (the anesthetic triad). Once reflexes such as patellar, palpebral, and corneal had ceased, an additional anesthetic dose was applied for the induction of euthanasia by anesthetic overdose. Before any further procedures were effected, death was verified by establishing the absence of vital signs and reflexes (corneal, eyelash, and rhythmic breathing). Blood was then collected by cardiac puncture to measure biomarkers of liver (ALT and AST) and kidney (BUN and creatinine) function and to conduct a hemogram. Transaminases, BUN, and creatinine assays were carried out from serum samples using commercial Ccromatest kits purchased from Linear Chemicals S.L.U. (Montgat, Spain), in accordance with the manufacturer’s instructions; and measurements were made using a URIT-8030 Automated Chemistry Analyzer. EDTA whole blood samples were used for hematological analysis. Total blood cell counts were performed using established URIT-3000Vet hematology equipment. Blood cell morphology and differential leukocyte counts were made on slides stained with Wright.

During the animals’ necropsies, photographic and weight records were collected from solid organs (spleen, liver, heart, and kidney), which were sectioned for subsequent immunohistochemical (heart tissue) and histopathological (kidney, liver, and spleen) analysis. These sections were placed into tubes containing 10% neutral formalin stabilized for 72 h, and then embedded in paraffin blocks. A fragment of cardiac tissue from the apical region of the left ventricle was collected in RNAlater^TM^ storage reagent (Sigma-Aldrich, St. Louis, MO, USA), for parasitemia determination by real-time polymerase chain reaction (qPCR). The remainder of the heart tissue was then divided by cross sectional cuts, with each half being randomly assigned for histologic or immunohistochemical analysis. To guarantee data blindness, all procedures (control of parasitemia, echocardiography, euthanasia, and sampling) were performed by independent veterinary personal unaware of which group the animal had been assigned to. The microscopic study was also performed blind by an expert pathologist. The animal remains were properly handled in accordance with the safety and environmental responsibility protocols established by the Universidad de Santander, and disposed of by incineration in accordance with the same.

### 2.6. Immunohistochemistry

The hearts were completely immersed into five tissue volumes of stabilized 10% neutral formalin solution, which was refreshed after 24 h. After one week, the tissues were placed into cassettes for embedding. The dehydration, clearing, and impregnation processes were carried out in a Leica TP1020 tissue processor (Leica Biosystems, Nussloch, Germany), loading the stations with 10% formaldehyde (2 h), 70% isopropyl alcohol (2 h), 80% isopropyl alcohol (2 h), 90% isopropyl alcohol (2 h), three absolute isopropyl alcohol solutions (2 h each), three xylene solutions (1 h and 30 s for the first solution and 1 h for the others), and two of liquid paraffin (2 h each). For the paraffin embedding of the tissues, a HistoCore Arcadia modular embedding system (Leica Biosystems, Nussloch, Germany) was used. Each block was first put on ice and cut into 4 μm thick sections on a HistoCore MULTICUT microtome with high-profile blades (Leica Biosystems, Nussloch, Germany), then placed in a flotation bath with a temperature of 45 °C to deparaffinize the tissue and prepare fragments for deposition onto positively charged slides (Thermo Fisher Scientific 4951PLUS, Cleveland, OH, USA). First, antigen recovery was accomplished by heating at 58 °C for 1 h and 30 min. For tissue deparaffinization, the slides were immersed in 3 xylol solutions (at 5-min intervals), in a 50% isopropanol solution (15 immersions), and in tap water (15 immersions). Plates were immersed in a 6% H_2_O_2_ solution for 5 min to block endogenous peroxidases, and cleansed. Antigen recovery was carried out by immersing the slides twice into a solution of ethylenediaminetetraacetic acid (EDTA), first at 95 °C for 30 min, then at room temperature for 20 min. Each slide was then dipped in a Coplin staining jar containing TBS-T for 10 min, and gently dried with a cotton fiber towel. The tissue area was delimited with a hydrophobic marker (Macrosearch Liquid Repellent). The slides were completely covered with “Ultra V Block” reagent (Ultravision Quanto Detection System HRP DAB kit, Thermo Fisher Scientific, Cleveland, OH, USA) for 5 min, cleared with TBS-T, and dried to remove excess moisture before proceeding with the application of antibodies. For the immunohistochemical technique, 100 µL of the desired antibody was applied to each slide for 1 h at room temperature, and then washed with TBS-T. The antibodies were diluted with Antibody Diluent Ventana^®^ (Roche) as follows: 1:1000 for Anti TNF-α (Abcam Reference ab6671, rabbit polyclonal to TNF-α); 1:250 for Anti IFN-γ (Abcam Reference ab216642, rabbit polyclonal to IFN-γ); 1:200 for Anti-Nitric Oxide Inducible Synthase iNOS (Abcam Reference ab15323, rabbit polyclonal to iNOS); 1:1000 for Anti IL-4 (Abcam Reference ab9811, rabbit polyclonal to IL-4); and 1:250 for IL-10 (Abcam Reference ab217941, and rabbit polyclonal to IL-10). To amplify the primary antibody reaction, a Primary Antibody Amplifier Quanto reagent (Ultravision Quanto Detection System HRP DAB kit, Thermo Fisher Scientific, Cleveland, OH, USA) was applied, followed by incubation for 10 min and rinsing with TBS-T. For revealing, HRP Polymer Quanto reagent (Ultravision Quanto Detection System HRP DAB kit, Thermo Fisher Scientific, Cleveland, OH, USA) and diaminobenzidine (DAB) (provided in the kit) was applied, following the manufacturer’s instructions. Finally, the slides were stained with hematoxylin-eosin. The process was completed with the adhesion of a 24 mm × 60 mm coverslip on each treated specimen. Brain, tonsils, and hearts from rats infected with *T. cruzi* were used in a standardized immunohistochemistry technique. An individual slide was used for each antibody to avoid possible contamination or cross-reaction.

For reading, the slides were scanned on a Ventana DP 200 whole-slide scanner (Roche Diagnostics, Rotkreuz, Switzerland), with the immunoreactivity calculation performed using free QuPath v. 0.2.3 software for digital pathological image analysis [13]. For this purpose, a project and a pixel classifier (px) were created with the following parameters: (i) Moderate resolution (2.00 µm/px); (ii) channel DAB; (iii) Gaussian prefilter; (iv) smoothing sigma 1; and (v) Threshold 0.25 (threshold value, positive results ≥ 0.25), defining the region of interest (ROI) as all cardiac tissue present on the slide. The results were expressed as a global percentage of immunoreactivity of the studied antibody.

### 2.7. DNA Extraction and Real Time PCR

Parasite loads in heart tissue were quantified post mortem by qPCR. For this procedure, parasite DNA was extracted using a commercial genomic DNA extraction kit (Invitrogen Life Technologies—Thermo Scientific, Waltham, MA, USA), in accordance with the manufacturer’s instructions. The obtained elute was quantified via Nanodrop 2000 (Thermo Scientific), and the purity and integrity of the DNA was verified in a 1.2% agarose gel. For parasite quantification in tissue by qPCR, the DNA extracted from cardiac tissue was amplified following the method described by Molina-Berríos [14], for identification of *T. cruzi* satellite DNA (GenBank accession number MH884804.1), employing Cruzi I: 5′-AST CGG CTG ATC GTT TTC GA-3′ and Cruzi II: 5′AAT TCC TCC AAG CAG CGG ATA-3′, as forward and reverse primers, respectively; and Cruzi III: 6FAMCAC ACA CTG GAC ACC AAMGBNFQ, as aTaqMan probe (5′Fluorescent label 6-FAM and 3′ Quencher MGB) acquired from Applied Biosystems (Applied Biosystems, Beverly, MA, USA). Cycle reactions were carried out in a Step One Plus^TM^ 3 (Applied Biosystems, Beverly, MA, USA) under the following conditions: one initial cycle of denaturation at 2 min and 50 °C and 10 min at 95 °C, followed by 35 cycles of denaturalization (15 s at 95 °C), and annealing and extension (60 s at 50 °C).The borderline Ct (Cycle threshold) of positivity was defined by preparing a standard curve via serial dilutions of 1:10, starting from a pure culture of *T. cruzi* (strain Sylvio-X10/4 TcI), and taking DNA points at 0.1; 1.0; 10; 100; and 1000 parasites. All the experiments were carried out in duplicate and performed at least three times independently.

### 2.8. Statistical Analysis

The clinical, hematological, and biochemical parameter data obtained were analyzed using GraphPad Prism software (San Diego, CA, USA). In this process, a descriptive analysis of the variables was carried out, estimating the frequency and dispersion measures for the quantitative variables, and proportions for qualitative variables. The results were expressed as means ± standard error of the mean (S.E.M.). The normality of the continuous variables was evaluated using the Shapiro–Wilk test. The normality of the continuous variables was evaluated using the Shapiro–Wilk test. For comparison between infected groups (positive control and experimental groups) and negative control, the one-way ANOVA test was employed, followed by Dunnett’s test; taking statistically significant differences to be *p* < 0.05. This statistical approach was applied for analysis of hematological (except polymorphonuclear (PMN) count cells), and biochemical results. For comparison between groups, the two-way ANOVA test was performed, with a post hoc analysis using Tukey, and taking significant differences to be *p* < 0.05. The results of the relative weight organ, ultrasound, and immunohistochemical assays were analyzed with a two-way ANOVA test, and a post hoc analysis using Tukey, taking significant differences to be *p* < 0.05.

## 3. Results

### 3.1. Chemical Compositions of L. alba Fractions

In the present study, EOs obtained by the steam distillation technique from fresh and mature carvone-chemotype *L. alba* leaves were fractionated under reduced pressure for enrichment in the bioactive terpene limonene. The relative composition of the EOs and their fractions, and the linear retention indices obtained by gas-coupled mass spectrometry, are presented in Table 2. In this work, the 90%-limonene fraction (F1) isolated from carvone-chemotype *L. alba* EOs was chosen as the base compound for the formulation of the two studied phytotherapies, LIMOX and LIMOXBNZ, as described in Table 2.

### 3.2. Induction of Experimental Chronic T. cruzi Infection in Wistar Rats

Successful induction of infection was verified by the presence of circulating parasites as observed by microscopic analysis, between 8 d.a.i. and 40 d.a.i. The levels of parasitemia between the groups of infected animals did not exhibit significant differences during the follow-up period (*p* < 0.05). Likewise, the onset and end of parasitemia demonstrated similar behavior between groups. The establishment of CCC was corroborated at 67 d.a.i. by the presence of echocardiographic characteristics of dilated cardiomyopathy (during the second ultrasound analysis) in infected animals. These cardiac imaging results were compared with the baseline established for the animals prior to infection (first ultrasound analysis). Thus, this period (67 d.a.i,) was defined as the onset of chronic disease, and therefore as the start date for administration of the oral treatment schemes.

### 3.3. Cardioprotective Activity

**Figure 1:**
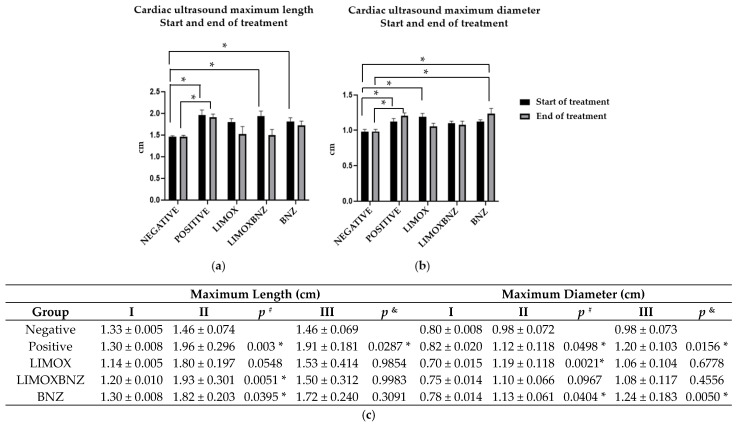
Echocardiography of Wistar rats infected with *Trypanosoma cruzi*. (**a**) Cardiac ultrasound maximum length start and end of treatment; (**b**) Cardiac ultrasound maximum diameter start and end of treatment; (**c**) Cardiac silhouette measurements via ultrasound (maximum length or ML and maximum diameter or MD) at the first (I), second (II), and third (III) echocardiography: (I) one day prior to infection; (II) at the start date of the therapies; and (III) at the end date of the treatments; *p* ^#^ compared with negative control measure from ultrasound II; *p* ^&^ compared with negative control measure from ultrasound III; * statistically significant difference at *p* < 0.05; Negative: untreated and uninfected animals; Positive: untreated and infected animals; LIMOX: infected animals treated with a mixture of an essential oil fraction of *L. alba* carvone chemotype enriched in limonene (68.9 mg/kg/day) and with added caryophyllene oxide (Sigma-Aldrich) (70 mg/kg/day); LIMOXBNZ: infected animals treated with LIMOX plus benznidazole (7.9 mg/kg/day); BNZ: infected animals treated with benznidazole (100 mg/kg/day). Data are representative of six independent experiments and values are expressed in mean ± SEM.

Following the completion of the treatment regimens (at 98 d.a.i.), cardiac effects were evaluated by echocardiography, recording the changes in the cardiac silhouette (maximum length or ML and maximum diameter or MD) at the third ultrasound analysis. Over this time interval, the results obtained showed no significant variations in either parameter for animals belonging to the negative control group. In contrast, animals infected and left untreated (positive control) and those infected and treated with BNZ (reference group) exhibited a significant increase in MD (*p* = 0.0156 and *p* = 0.0050, respectively). In fact, the animals assigned to these two groups were the only ones which demonstrated effects upon clinical parameters; exhibiting decay, dehydration, piloerection, and brown discharge in the conjunctiva (a stress indicator). In contrast, a reversion of both cardiac measures (ML and MD) to values similar to those found in uninfected subjects (negative control, *p* > 0.05) were evidenced in those infected animals which received the LIMOX and LIMOXBNZ therapies (with statistically significant differences when compared to the positive control, *p* < 0.05) (Figure 1).

**Figure 2:**
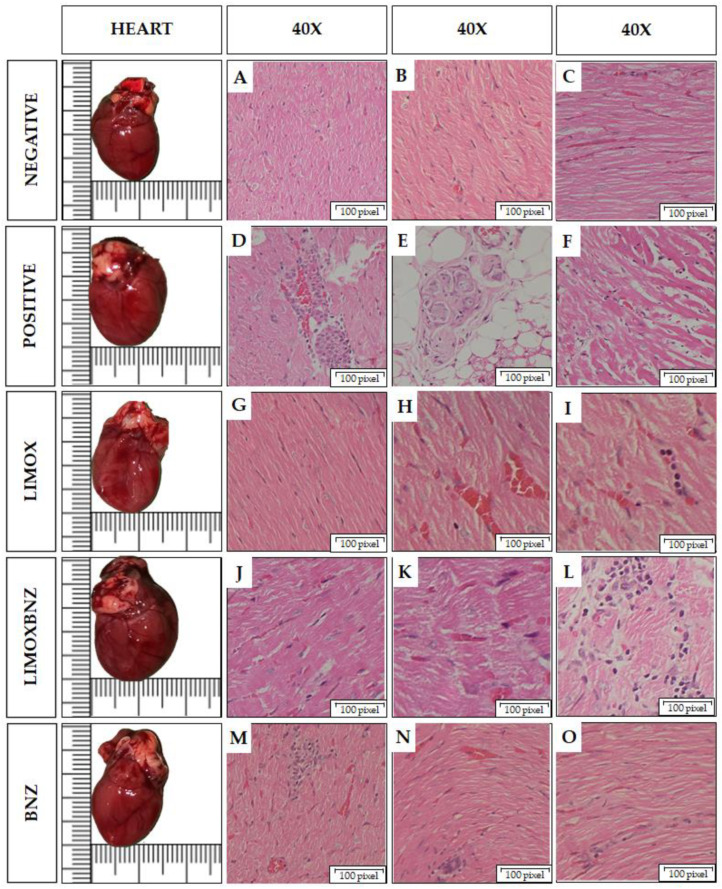
Macro and microscopic findings in the hearts of Wistar rats infected with *Trypanosoma cruzi*. Histological images taken using a 40× objective on Hematoxylin and Eosin stained tissue sections. Negative: untreated, uninfected animals; Positive: untreated, infected animals; LIMOX: infected animals treated with an essential oil fraction of *Lippia alba* carvone chemotype enriched in limonene (68.9 mg/kg/day) and with added caryophyllene oxide (Sigma-Aldrich) (70 mg/kg/day); LIMOXBNZ: infected animals treated with LIMOX and benznidazole (7.9 mg/kg/day); BNZ: infected animals treated with benznidazole (100 mg/kg/day). (**A**–**C**): Normal heart tissue. (**D**). Large focal inflammatory infiltrate in myocardium with lymphocytes, histiocytes, and plasma cells. (**E**). Aggregate of cardiac plexus neurons, surrounded by lymphocytic infiltrate. (**F**). Multifocal infiltrate with minimal angiogenesis and loose, elongated, and sinusoidal fibers with fibrotic process. (**G**). Small histiocytic inflammatory infiltrate. (**H**). Apparent reparative process. (**I**). Lymphocytic inflammatory infiltrate with minimal fibrosis. (**J**). Loose and elongated fibers, with histiocytic lymphocyte inflammatory infiltrate. (**K**). Mild lymphohistiocytic infiltrate. (**L**). Diffuse linear inflammatory infiltrate, with minimal fibrosis. (**M**) Focal of inflammatory infiltrate in plasma lymphocytoid, loose and elongated fibers. (**N**). Foci of lymphocytic inflammatory infiltrate; loose, elongated fibers. (**O**). Focal of inflammatory infiltrate of lymphocytes and plasma cells; dilated, loose, and elongated fibers with a sinuous appearance; with fibrosis. Figures are representative of six independent experiments.

Post mortem macroscopic and microscopic (histopathology) heart observations are presented in Table 3 and Figure 2. The successful induction of the experimental cardiomyopathy model was confirmed by bulging and dilated forms, especially in the left ventricles, of the hearts in the positive control group (observed in 100% of the animals). Consistently, in these animals, histopathology evidenced: multiple foci of inflammatory infiltrate with cell diversity (predominantly lymphocytes, plasma cells, and histiocytes); damage to the myocardial tissue, the atria-ventricle junction sites, and the neurons of the cardiac plexus [10,15].

In infected animals submitted to the array of treatments, the best performance in terms of protection against cardiac damage induced by the *T. cruzi* infection was observed in LIMOX treated animals, followed by LIMOXBNZ (Table 3 and Figure 2). In contrast, among the experimental groups, major effects upon the heart structure were apparent in the BNZ group, whose animals presented macroscopic and microscopic features very similar to infected and untreated rats (positive control).

### 3.4. Immunomodulation

**Figure 3:**
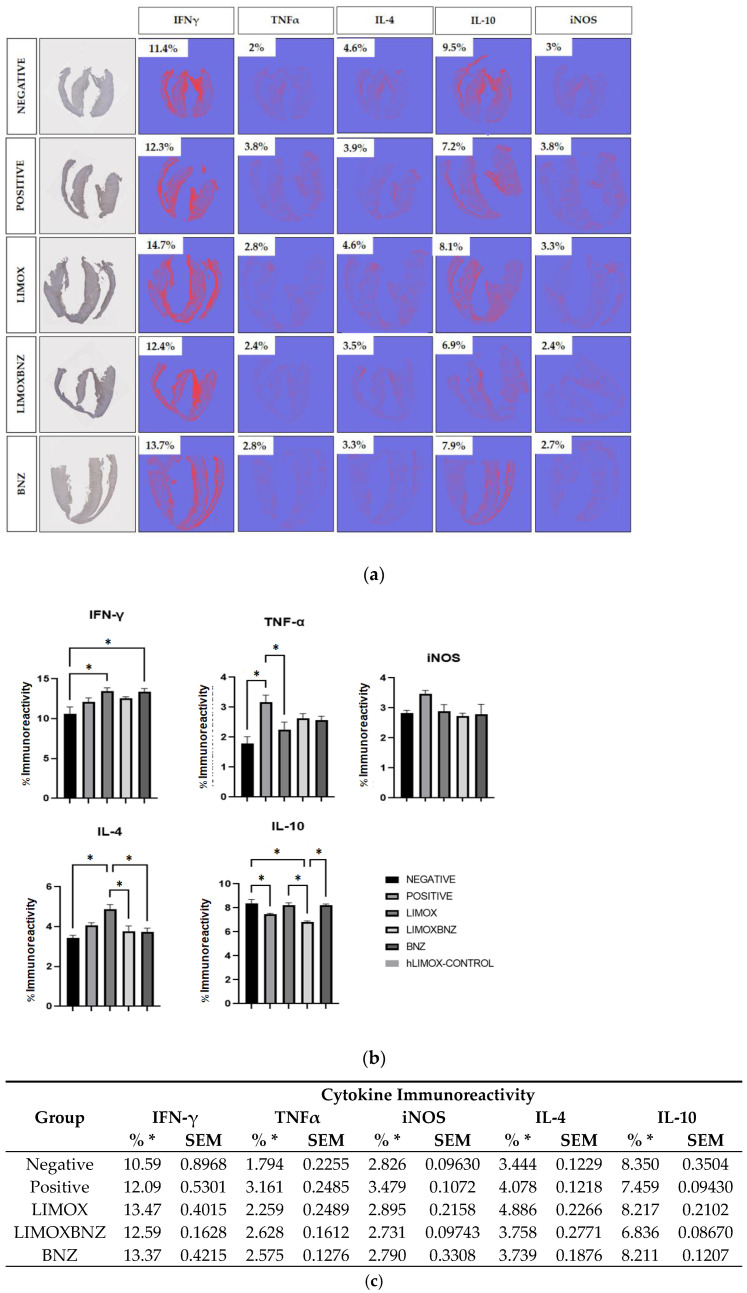
Immunohistochemical analysis of cardiac tissue (**a**) Percentages of immunoreactivity obtained on cross sectional cuts of heart tissue by specific antibodies for Interferon gamma (IFN-γ), Tumor Necrosis Factor alpha (TNF-α), Interleukin (IL)-4, IL-10, and inducible Nitric Oxide Synthase (iNOS); (**b**,**c**) Comparison of immunoreactivity percentages within groups. Negative: untreated and uninfected animals; Positive: untreated and infected animals; LIMOX: infected animals treated with an essential oil fraction of *L. alba* carvone chemotype enriched in limonene (68.9 mg/kg/day) with added caryophyllene oxide (Sigma-Aldrich) (70 mg/kg/day); LIMOXBNZ: infected animals treated with LIMOX and benznidazole (7.9 mg/kg/day); BNZ: infected animals treated with benznidazole (100 mg/kg/day). All *p* values were calculated by comparison within groups. * *p* ≤ 0.05. Data are representative of six independent experiments and values are expressed in mean ± SEM.

Immunohistochemical analysis revealed a very distinct cytokine profile between uninfected (negative control) and infected animals (positive and experimental groups), as well as between untreated rats and those subjected to some types of therapy (Figure 3). In this regard, animals without *T. cruzi* infection exhibited the lowest levels of pro-inflammatory cytokines (IFN-γ and TNF-α), and the pro-oxidant marker, iNOS. On the other hand, as a result of infection, a significant increase of TNF-α was observed in infected and untreated rats with a concomitant reduction of the anti-inflammatory IL-10. With respect to treatments, LIMOX and BNZ stimulated a significant increase in IFN-γ immunoreactivity, compared to the negative control; with LIMOX as the only therapy able to reduce TNF-α levels in a significant manner (compared to positive control). Interestingly, the highest levels of the anti-inflammatory cytokine IL-4 were exhibited by the infected animals treated with LIMOX, at a significant difference with respect to the other groups (negative control, LIMOXBNZ, and BNZ). Correspondingly, this treatment was the only therapy able to reestablish the IL-10 levels to those found in uninfected animals. Finally, the iNOS analysis revealed a notable reduction of this oxidant marker (similar to the uninfected model) given by the experimental treatments (LIMOX, LIMOXBNZ, and BNZ), in comparison with the infected and untreated animals (Figure 3).

### 3.5. Trypanocidal Effect

*T. cruzi* DNA quantification (qPCR) analysis demonstrated that animals from the negative and positive control groups exhibited 0% (Ct ≥ 32) and 100% positivity (Ct < 32), respectively; while the applied therapies demonstrated a range of trypanocidal effectivity ranging from 66% (LIMOXBNZ) to 83% (LIMOX and BNZ) (Table 4).

### 3.6. Toxicity

**Figure 4:**
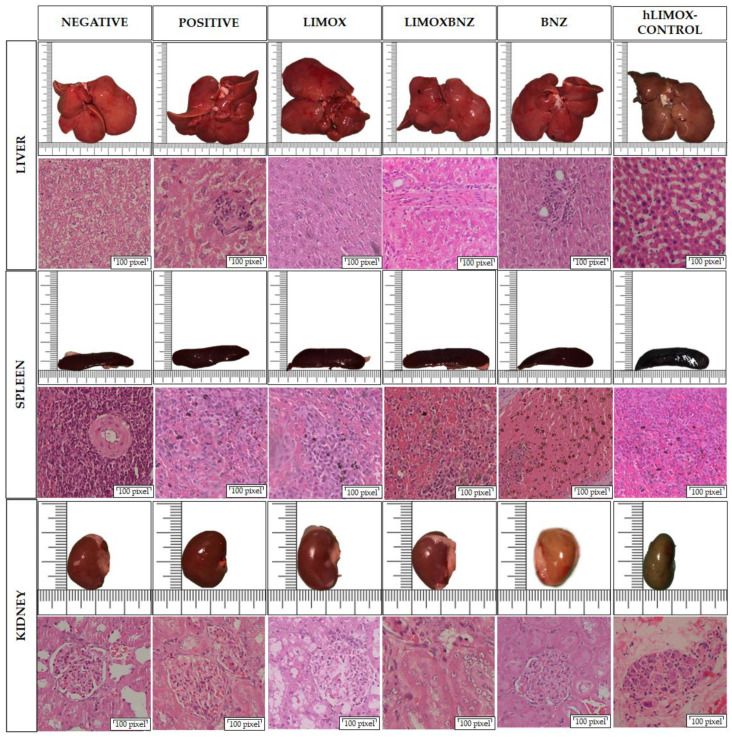
Organ toxicity assessment. Histological images taken using a 40× objective on Hematoxylin and Eosin stained tissue sections. Negative control: untreated, uninfected animals; hLIMOX-Control: not infected animals treated with higher doses of an essential oil fraction of *Lippia alba* carvone chemotype enriched in limonene (170 mg/kg/day) and with added caryophyllene oxide (Sigma-Aldrich) (70 mg/kg/day); Positive control: untreated, infected animals; LIMOX: infected animals treated with a mixture of an essential oil fraction of *Lippia alba* carvone chemotype (enriched in limonene) (68.9 mg/kg/day) and with added caryophyllene oxide (Sigma-Aldrich) (70 mg/kg/day); LIMOXBNZ: infected animals treated with LIMOX plus benznidazole (7.9 mg/kg/day); BNZ: infected animals treated with benznidazole (100 mg/kg/day). Figures are representative of six independent experiments.

**Figure 5:**
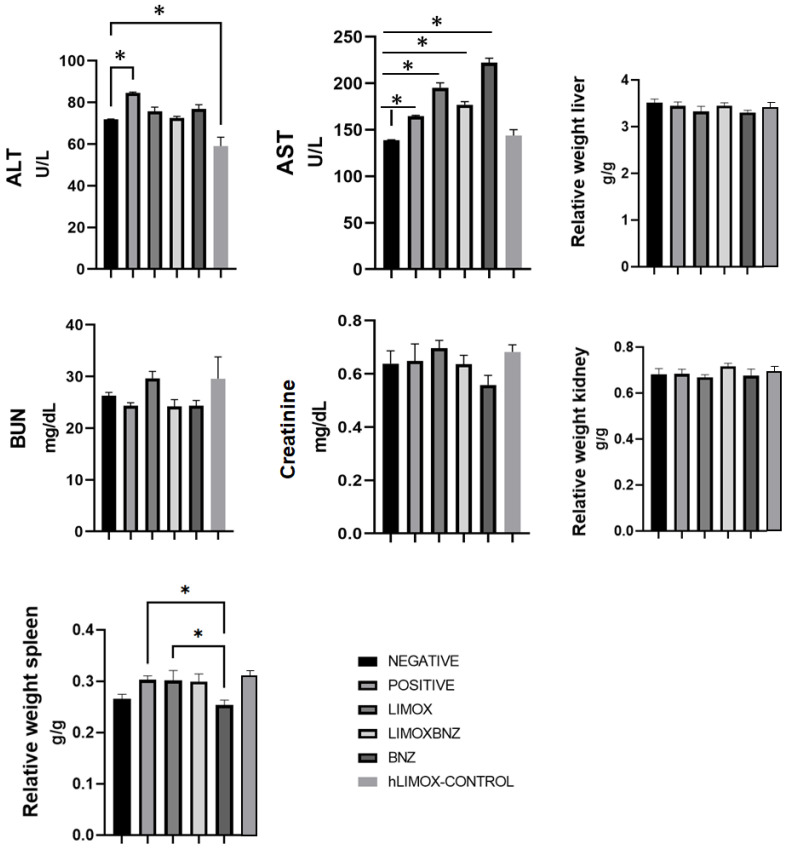
Toxicity of the tested therapies in the liver and spleen. ALT: Alanine aminotransferase; AST: Aspartate aminotransferase; BUN: Blood urea nitrogen. * *p* ≤ 0.005 compared to the Benznidazole group. Negative control: untreated, uninfected animals; hLIMOX-Control: not infected animals treated with higher doses of an essential oil fraction of *Lippia alba* carvone chemotype enriched in limonene (170 mg/kg/day) and with added caryophyllene oxide (Sigma-Aldrich) (70 mg/kg/day); Positive control: untreated, infected animals; LIMOX: infected animals treated with a mixture of an essential oil fraction of *Lippia alba* carvone chemotype (enriched in limonene) (68.9 mg/kg/day) and with added caryophyllene oxide (Sigma-Aldrich) (70 mg/kg/day); LIMOXBNZ: infected animals treated with LIMOX plus benznidazole (7.9 mg/kg/day); BNZ: infected animals treated with benznidazole (100 mg/kg/day). * *p* ≤ 0.05. Data are representative of six independent experiments and values are expressed in mean ± SEM.

**Figure 6:**
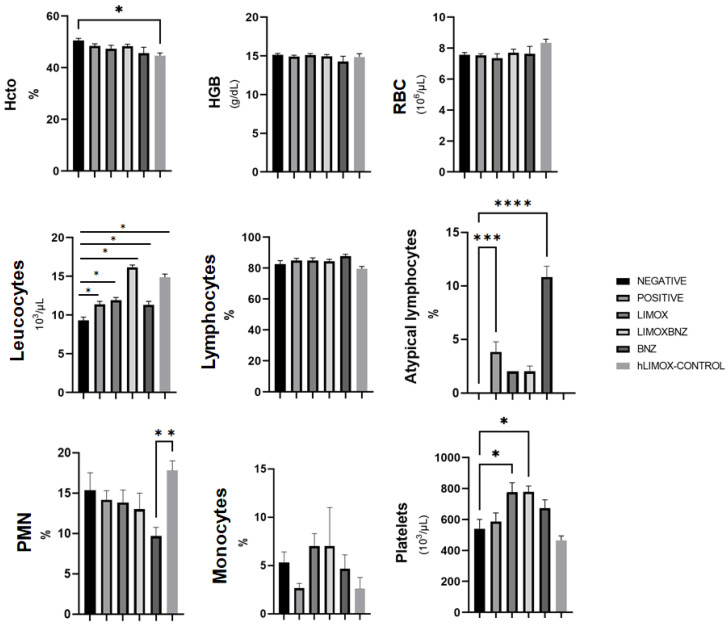
Hematological findings in Wistar rats infected with *Trypanosoma cruzi*. Hcto: Hematocrit; HGB: Hemoglobin; Negative: untreated, uninfected animals; hLIMOX-Control: not infected animals, treated with higher doses of an essential oil fraction of *Lippia alba* carvone chemotype enriched in limonene (170 mg/kg/day) and with added caryophyllene oxide (Sigma-Aldrich) (70 mg/kg/day); Positive: untreated, infected animals; LIMOX: infected animals treated with an essential oil fraction of *Lippia alba* carvone chemotype (enriched in limonene) (68.9 mg/kg/day) with added caryophyllene oxide (Sigma-Aldrich) (70 mg/kg/day); LIMOXBNZ: infected animals treated with LIMOX plus benznidazole (7.9 mg/kg/day); BNZ: infected animals treated with benznidazole (100 mg/kg/day). * *p* < 0.05 compared to the negative control group; ** *p* < 0.05 compared to the BNZ group; *** *p* < 0.005 compared to the negative control group **** *p* < 0.0001 compared to the negative control group. Data are representative of six independent experiments and values are expressed in mean ± SEM.

The toxicity of each treatment was assessed by histopathological analysis of the kidney, liver, and spleen. No abnormalities were observed in the macro and microscopic analyses of the organs obtained from negative control animals, as well as from not infected rats treated with higher doses of LIMOX (hLIMOX-Control). In contrast, large mononuclear-type inflammatory infiltrates were present in the livers of infected and untreated animals (positive control) (Figure 4). Minimal evidence of inflammation in this organ was also discovered in infected rats treated with phytotherapeutical regimens (Table 5). Correspondingly, this finding was accompanied by significantly increased AST levels (compared with the negative control), however, heightened levels of this transaminase were also observed in animals treated with BNZ (Figure 5 and Figure 6).

Additionally, splenomegaly was evident in 16.6% of the animals in the LIMOXBNZ-treated experimental group and in 50% of the animals belonging to the LIMOX-treated and positive control groups (Figure 4). In contrast, a significant reduction in the relative weight of this organ was evident in animals subjected to BNZ treatment (Figure 4 and Table 5). In addition, therapies which included that same compound (i.e., BNZ or LIMOXBNZ) induced the presence of hemosiderophages in the spleens of some of the treated animals (16.6%) (Figure 3). In a similar manner, kidney toxicity was apparent in 16.6% of the rats treated with BNZ, presenting as a pale brown color in the macroscopic analysis (Figure 3), but without altering the histopathological or biochemical parameters in the organ. With respect to BUN, a statistically significant elevation in this marker was observed in the LIMOX group compared to the reference treatment (BNZ) (*p* = 0.0048), but with no difference in creatinine values (Figure 5).

With respect to hemogram parameters, thrombocytosis was associated with the LIMOX and LIMOXBNZ therapies, and was statistically significant in relation to the other treatments (*p* = 0.018 and *p* = 0.017, respectively). Regarding the leukocyte count, all infected groups presented a tendency (not statistically significant) towards higher counts than uninfected animals. In addition, the presence of atypical lymphocytes could be observed in the rats treated with BNZ, with a significant difference in relation to the other groups. The remaining hematological parameters did not exhibit significant alterations (Figure 6). An evaluation of the phytotherapies’ toxicity shows that high doses of LIMOX (2.5 times the therapeutic dose) applied to not infected animals (hLIMOX-Control) caused minor signs of toxicity, particularly a change in the color of the kidney (exhibiting a pale brown shade in the macroscopic analysis), in 16.6% of the treated rats. Likewise, a statistically significant peripheral blood neutrophilic leukocytosis was also observed in this same group of animals.

## 4. Discussion

Chagas heart disease (CHD) is characterized by dilated cardiomyopathy that affects the atria, ventricles, conduction system, and autonomic nervous system (ANS) [16]. In the pathogenesis of CHD, various mechanisms are implicated such as: the persistence of the parasite; denervation; both microvascular and endothelial dysfunction; a persistent and exacerbated immune response (via the imbalance between pro-inflammatory and anti-inflammatory cytokines) or even autoimmunity [17]; as well as the induction of a permanent oxidative stress caused by both reactive nitrogen species (RNS) and reactive oxygen species (ROS), which in turn directly affects the structure and function of the respiratory chain of cardiac tissue [18]. All of these factors combine to prevent the correct remodeling of heart tissue (functional tissue is replaced by non-functional fibrotic tissue), ultimately leading to heart failure, for which the only treatment option is transplantation [3].

Hence, it is urgent that new therapeutic alternatives be found to eliminate the parasite, control inflammation, and ameliorate cardiac damage, as well as minimizing the harmful side effects in human treatment posed by the current therapeutic options. In this sense, phytoderivatives obtained from aromatic plants represent a highly diverse platform for the discovery of bioactive compounds. In our group, promising in vitro trypanocidal activity was reported for the major terpenes of *L. alba* EOs [8], limonene and caryophyllene oxide; with limonene being the most effective compound against all parasite forms (IC_50_ of 9.0, 28.7, and 41.8 µg/mL for amastigotes, trypomastigotes, and epimastigotes, respectively) [8]. This activity was also exhibited in in vivo models applied to Wistar rats with chronic *T. cruzi* infection, which were treated with synergistic fractions of these EOs enriched in limonene and caryophyllene oxide [10]. Recently, in vitro immunomodulatory properties on macrophages infected with *T. cruzi* were also ascribed to these phytoderivatives [9].

In order to clarify the potential role of certain relevant anti- and pro-inflammatory cytokines and oxidative markers in the trypanocidal and cardioprotective activities observed for *L. alba* EO, the present study assessed the behavior of these markers in an in vivo model of *T. cruzi* chronic infection induced in Wistar rats. In infected and untreated animals (positive control), CHD was verified by the appearance of a statistically significant enlargement of cardiac silhouette parameters (MD and ML) at 67 d.a.i (*p* < 0.05, compared to the uninfected group). Histological findings observed in the animals belonging to the positive control group confirmed the success of CCC induction; findings such as: damage to cardiac tissue, mainly in the form of loose, elongated, and sinusoidal fibers in the myocardium; the presence of multifocal and diffuse mixed inflammatory infiltrates (predominantly of the lymphohistiocytic type); and foci of lymphohistiocytic infiltrate in cardiac plexus neurons [3,19]. At that time, continuous once-daily oral treatments with the three studied schemes (LIMOX, LIMOXBNZ, and BNZ), were administrated for 31 days.

The results showed that the treatment comprised of a mixture of limonene-enriched fraction of *L. alba* EOs and caryophyllene oxide (LIMOX) demonstrated the best performance in restoring the normal shape of the heart compared to the other therapies trialed (LIMOXBNZ and BNZ). As such, the heart dimensions of animals belonging to the LIMOX group exhibited the most reduced ML and MD measurements among infected animals, even to the point of regression to values similar to those of the uninfected control (negative). Not unexpectedly, positive control group rats (infected and untreated) presented the most notable bulging and dilated shape, followed closely those of the BNZ and LIMOXBNZ groups.

Histologically, animals treated with LIMOX evidenced very few inflammatory foci and minimal fibrosis in the heart tissue, with similar characteristics to those of the negative control, in the whole-slide scanning analysis. These results could be attributable to the trypanocidal and cardioprotective effects previously ascribed to limonene and caryophyllene oxide [8,10]. In contrast, somewhat more inflammatory foci were discovered in the tissues of the animals treated with LIMOXBNZ, in whom the signs of fibrosis were also more evident. These adverse effects could be due to the subtherapeutical BNZ doses present in the mixture, which may promote a pro-inflammatory response [10,15]. In a similar manner, animals treated with the conventional intervention (BNZ) exhibited multiple inflammatory foci in the heart, with greater cell variety (particularly lymphocytes, histiocytes, plasma cells, and monocytes), a greater area of fibrous tissue, larger cardiomyocytes, and necrosis.

In order to assess, in a histological context, the cardiac immune response modulated by chronic *T. cruzi* infection and by the therapies trialed, the present study applied an immunohistochemical technique targeting a variety of antigens relevant to CCC pathogenesis using Qupath software; an open-source whole-slide image analysis tool [13]. The results showed that the highest percentages of immunoreactivity for TNF-α and iNOS were present in the infected and untreated animals of the positive control group; as were significantly increased levels of IFN-ɣ. These findings were accompanied by the impairment of the anti-inflammatory IL-4. In this regard, IFN-ɣ regulates over a thousand genes by activating Janus tyrosine kinase (JAK) and phosphorylation of the transducer, and serving as an activator of the transcription 1 (STAT-1) pathway. This latter induces the transcription of TNF-α, interferon-inducible factor 1 (IRF1), and iNOS, among other inflammatory cytokines and chemokines [20]. Likewise, in the context of Chagas disease, IFN-γ acts synergistically with TNF-α through the activation of the nuclear transcription factor NF-kB for the positive regulation of iNOS expression; producing high levels of nitric oxide and RNS [21]. This phenomenon represents the activation of general trypanocidal mechanisms, such as the production of reactive species, through the induction of mitochondrial ROS and NADP oxidases [20,22]. These reactive species promote the production of the peroxynitrite anion, a strong oxidant mechanism that arises as a strategy for the elimination of the parasite; causing morphological disruption, severe alterations in its metabolism, calcium homeostasis, and trypanothione depletion [23,24].

Coherently, in this study, the most trypanocidal therapies LIMOX and BNZ (with 83% of parasitological cure in cardiac tissue assessed by qPCR), were accompanied by the highest IFN-ɣ immunoreactivity. In this regard, it was hypothesized that in *T. cruzi* infection, the elevated levels of this cytokine could be a double-edged sword; since despite its recognized role in parasite tissue clearance and as an antifibrotic agent [25], an excess of IFN-ɣ production could cause serious damage to cardiac tissue [25]. Nevertheless, an apparent regulatory mechanism was observed in animals subjected to LIMOX therapy (though not in the case of BNZ), represented by an increase in IL-4 production accompanied by restoration of IL-10 levels and lower percentages of TNF-α (compared to the other experimental groups, *p* < 0.05) and iNOS (whose levels did not exhibit statistically significant differences from the negative control, *p* > 0.05).

These differences in the tissue profile of pro- and anti-inflammatory mediators could explain the significant macroscopic and microscopic differences observed in the cardiac architecture between the LIMOX and BNZ groups; in which only animals treated with the terpene mixture presented regression in diameter measurements of the heart silhouette and significant reduction in inflammatory infiltrates or foci with fibrosis. In this context, it is known that TNF-α can induce collagen synthesis and fibrosis, thus contributing to the loss of cardiomyocyte contractility and its replacement by fibrotic tissue [25]. In addition, the same substance can be involved in the development of heart failure through apoptosis and induction of iNOS, with the consequent production of nitric oxide, which exerts a very strong inotropic effect [25]. These findings have been consistently documented in patients who expired from CCC [26]. Likewise, the expression of IL-10 and IL-4 has been linked to the improvement of cardiac function, as determined by the values of the left ventricular ejection fraction and the ventricular diastolic diameter [26]; factors which constitute a possible immunomodulatory tolerance mechanism which could potentially prevent cardiac damage. In this respect, IL-10 is considered a very important regulatory cytokine, and its production is associated with a better prognosis in chronic CHD, suggesting a protective effect for the Th1 response [26,27,28,29].

It is worth mentioning that these compensatory mechanisms represented by the stimulation of IL-4 and IL-10 secretion were not observed in the combined therapy of LIMOX and BNZ (LIMOXBNZ), in which both IFN-γ and TNF-α were slightly increased without a significant anti-inflammatory response. These findings suggest that BNZ, even in subtherapeutical doses, causes an antagonist effect on the Th1 response triggered by LIMOX therapy. These results align with those of Quintero et al. [9] who reported that therapies composed of BNZ, alone or in combination with *L. alba* fractions (rich in limonene and citral/caryophyllene oxide), impaired IL-4 secretion by *T. cruzi*-infected macrophages.

In the same work, Quintero et al. [9] showed that the synergistic combination of *L. alba* fractions rich in limonene and citral/caryophyllene oxide produced a significant reduction of the pro-inflammatory cytokines (IFN-γ, IL-2, and TNF-α), with a concomitant increase of the anti-inflammatory cytokines (IL-4 and IL-10), in the extracellular media of the infected macrophages [9]. Although both studies (the aforementioned as well as the current work) show evidence of an immunomodulatory effect (reduction of all pro-inflammatory cytokines with significant elevation of IL-4) by fractions derived from *L. alba* EOs enriched in limonene and caryophyllene oxide, a significant disagreement exists with respect to the behavior of IFN-γ [9]. In this context, the elevated IFN-γ levels observed only in the present in vivo study could be explained by additional compensatory cellular mechanisms converging in the global response of the innate immune system, which can be uniquely perceived in a context of cardiac tissue [25].

Regarding the toxicity of the therapies trialed, slight hepatomegaly accompanied by mild to moderate microscopic signs of periportal inflammation were reported in a percentage (16.6%) of animals belonging to the groups treated with both studied phytotherapies (LIMOX and LIMOXBNZ). These morphological features were correlated with elevated levels of AST transaminase (*p* < 0.05) in these same rats. On the other hand, kidney function assessed by biochemical, morphological, and histopathological analysis did not exhibit any significant alterations among control and experimental groups. Nevertheless, a statistically significant elevation of BUN levels was found in infected rats treated with LIMOX when compared to the reference treatment (BNZ) (*p* ≤ 0.005). In addition, 16% of the animals treated with BNZ presented a macroscopic alteration in the color of the kidney (pale brown).

With reference to an effect on spleen architecture, differential responses were observed among therapies. Specifically, splenomegaly was present in the positive control animals and in those treated with both LIMOX and LIMOXBNZ, while BNZ caused a reduction in the size of this organ. This hyposplenism could be correlated with the deleterious effect on adequate balancing of the defense response, as observed herein, in animals treated with the reference therapy [30].

In hemogram analysis, a significant thrombocytosis (*p* < 0.005) was reported for both the LIMOX and LIMOXBNZ therapies. Interestingly, platelet counts in peripheral blood have been inversely associated with disease severity in patients with CCC [31]. In this regard, platelets play an important role in immune response, including protective functions via the release of chemokines that attract and activate leukocytes and, at the same time, platelet surface molecules such as P-selectin and GPIIB/IIIa (Glycoprotein IIb/IIIa) [31]. Likewise, atypical lymphocytes were observed in the peripheral blood smear analysis of all infected animals (experimental groups and positive control) with the highest percentage of these reactive cells in BNZ-treated rats (10.8% for BNZ vs. 3.8% for positive control and 2% for LIMOXBNZ and LIMOX). These cells are produced after a strong process of antigenic stimulation and stress, being classified as a nonspecific response to stimulus or as precursors of memory T and B cells [31]. However, a low percentage of these cells can normally be found in peripheral blood (2–6%) [32], thus the abnormally elevated percentages observed in BNZ therapy could be a reflex to its toxicity.

## 5. Conclusions

This research contributes to clarifying, in a chronic CHD model in Wistar rats, immunomodulation as a possible trypanocidal and cardioprotective mechanism of LIMOX, a therapy based on a synergistic mixture composed of caryophyllene oxide and a limonene-enriched fraction derived from *L. alba* EO. This therapy showed clear benefits in controlling parasite load, apparently through a mechanism related to the enhancement of the nonspecific immune response mediated by high levels of IFN-γ. Remarkably, LIMOX also demonstrated high performance in controlling the progression of cardiac involvement in vivo, and even reversing the progression of dilated cardiomyopathy to levels similar to those found in uninfected animals. Correspondingly, a significant reduction in the severity of inflammatory foci and tissue damage was also confirmed by histopathological analysis, as well as greater tissue remodeling function. The cardioprotective effect observed via LIMOX treatment was correlated with a protective mechanism derived from an increase in levels of the anti-inflammatory interleukin, IL-4, with a concomitant decrease of TNF-α and a reestablishment of IL-10. Thus, LIMOX becomes an interesting compound for the development of a holistic alternative therapy for the treatment of the chronic phase of Chagas disease.

## Figures and Tables

**Table 1:** Therapeutic schemes administrated in *Trypanosoma cruzi*-infected Wistar rats.

Group	Inoculum (Tryp)	Vehicle (Sunflower Oil) *q. s. p*	*Lippia alba* Carvone- Chemotype EO: Limonene- Enriched Fraction (mg/Kg/day)	Caryophyllene Oxide (mg/Kg/day)	Benznidazole (mg/Kg/day)
Negative	-	100 µL	-	-	-
hLIMOX-Control	-	100 µL	170	70	-
Positive	2.5 × 10^5^	100 µL	-	-	-
LIMOX	2.5 × 10^5^	100 µL	68.9	70	
LIMOXBNZ	2.5 × 10^5^	100 µL	68.9	70	7.9
Benznidazole	2.5 × 10^5^	-	-	-	100

Tryp: metacyclic trypomastigote of *Trypanosoma cruzi* clone 338Cl2 TcI; EO: Essential oil; *q. s. p*: quantity sufficient provided.

**Table 2:** Main compounds present in the essential oils and their fractions obtained from *Lippia alba* (carvone chemotype). Taken and adapted with permission from Quimbaya et al. [10].

Compound	Linear Retention Indices		Relative Areas GC, % (Media, *n* = 3)			
	Column		Carvone Chemotype			
	DB-5	DB-WAX	EO	F1 [106 °C]	F2 [115 °C]	F3 [120 °C]
6-Methyl-5-hepten-2-one	986	1340	0.1	0.1	-	-
*p*-Cymene	1024	1274	0.1	0.5	1.1	-
Limonene	1030	1202	30.6	90.5	0.7	0.1
Terpinolene	1086	1284	0.3	0.9	1.5	-
*trans*-Dihydrocarvone	1202	1626	0.2	0.1	1.0	2.5
Carvone	1242	1736	51.2	1.5	69.9	86.6
Geranial	1270	1728	0.1	-	-	-
Piperitone	1342	1912	1.5	0.2	2.2	2.3
α-Copaene	1376	1492	0.4	0.1	2.9	3.1
β-Elemene	1390	1592	0.5	0.3	2.6	2.9
*trans*-β-Caryophyllene	1420	1600	0.3	-	0.2	0.4
α-Humulene	1454	1670	0.4	-	-	0.1
Germacrene D	1482	1712	0.3	-	0.1	0.2
Bicyclogermacrene	1496	1738	7.5	0.9	6.3	7.2
β-Bisabolene	1508	1730	0.4	-	-	-
Caryophyllene oxide	1582	1990	0.2	-	0.1	-

DB-5: Gas chromatography (60 m) column; DB-WAX: Gas chromatography (60 m) column; EO: Essential oils; F: fraction; GC: Gas chromatography. Data are representative of three independent experiments and the mean of three relative GC areas was reported for each compound.

**Table 3:** Macroscopic and microscopic findings in the hearts of Wistar rats after receiving treatments.

Parameter	Control Groups			Experimental Groups		
	Negative	hLIMOX-Control	Positive	LIMOX	LIMOXBNZ	BNZ
Color	Normal	Normal	Normal	Normal	Normal	Normal
Size	Normal	Normal	Cardiomegaly	Cardiomegaly	Cardiomegaly	Cardiomegaly
RW g/Kg (Ⴟ ± SEM)	400 ± 86	400 ± 30	402 ± 58	385 ± 30	410 ± 32	375 ± 34
Histopathology	Normal	Normal	Multiple foci of inflammatory infiltrate with cellular diversity and cardiac tissue damage	Heart tissue with minimal inflammatory foci and sporadic mild fibrosis	Heart tissue with mild inflammatory foci and mild fibrosis	Major cardiac damage with multiple fibrosis, cardiac fiber damage, and moderate to abundant inflammatory foci

RW: Relative weight; SEM: standard error of the mean; Ⴟ: mean; Negative: untreated, uninfected animals; hLIMOX-Control: not infected animals treated with higher doses of an essential oil fraction of *Lippia alba* carvone chemotype enriched in limonene (170 mg/kg/day) and with added caryophyllene oxide (Sigma-Aldrich) (70 mg/kg/day); Positive: untreated, infected animals; LIMOX: infected animals treated with an essential oil fraction of *L. alba* carvone chemotype enriched in limonene (68.9 mg/kg/day) and with added caryophyllene oxide (Sigma-Aldrich) (70 mg/kg/day); LIMOXBNZ: infected animals treated with LIMOX and benznidazole (7.9 mg/kg/day); BNZ: infected animals treated with benznidazole (100 mg/kg/day). Data are representative of six independent experiments and values are expressed in mean ± SEM.

**Table 4:** Quantification of parasite DNA by real-time PCR of heart tissue from Wistar rats infected with *Trypanosoma cruzi* and treated with 31 continuous once-daily oral doses.

	Control Groups		Experimental Groups		
Animal	Negative (Ct)	Positive (Ct)	LIMOX (Ct)	LIMOXBNZ (Ct)	BNZ (Ct)
1	34.833	29.855 *	38.161	33.000	38.540
2	37.341	27.391 *	29.294 *	37.937	38.598
3	38.096	26.325 *	38.545	30.839 *	32.581
4	33.399	25.425 *	37.726	38.579	31.197 *
5	35.845	27.240 *	36.843	30.323 *	33.536
6	34.347	23.875 *	35.895	33.124	38.785
Positivity (%)	0	100	17	33	17

Negative: untreated, uninfected animals; Positive: untreated, infected animals; LIMOX: infected animals treated with an essential oil fraction of *Lippia alba* carvone chemotype (enriched in limonene) (68.9 mg/kg/day) with added caryophyllene oxide (Sigma-Aldrich) (70 mg/kg/day); LIMOXBNZ: infected animals treated with LIMOX plus benznidazole (7.9 mg/kg/day); BNZ: infected animals treated with benznidazole (100 mg/kg/day). * Positive result with Ct value > 32. Data are representative of three independent experiments performed in duplicate and values are expressed as means.

**Table 5:** Macroscopic and microscopic findings in organs of Wistar rats after receiving treatments.

Organ/ Parameter	Control Groups			Experimental Groups		
	Negative	hLIMOX-Control	Positive	LIMOX	LIMOXBNZ	BNZ
Liver						
Color	Normal	Normal	Normal	Normal	Normal	Normal
Size *	Normal	Normal	Hepatomegaly (1/6)	Hepatomegaly (2/6)	Hepatomegaly (2/6)	Normal
RW g/Kg (Ⴟ ± SEM)	3.510 ± 198	3.500 ± 200	3.443 ± 210	3.327 ± 263	3.447 ± 159	3.303 ± 114
Histopathology	Normal	Normal	Lymphocytic mononuclear infiltrate (4/6)	Periportal lymphocytic mononuclear infiltrate (1/6)	Large dilation of the central vein of the lobule. Blood returns from the superior vena cava: volume and pressure overload (1/6)	Minimal lymphocytic mononuclear infiltrate around the portal (1/6)
**Spleen**						
Color	Normal	Normal	Normal	Normal	Normal	Normal
Size *	Normal	Normal	Splenomegaly (3/6)	Splenomegaly (3/6)	Splenomegaly (1/6)	Hyposplenism (3/6)
RW g/Kg (Ⴟ ± SEM)	266 ± 20	260 ± 30	303 ± 19	301 ± 47	300 ± 36	253 ± 25
p *	0.872	0.910	0.035	0.043	0.053	1
Histopathology	Normal	Normal	Normal	Normal	Hemosiderophages Observed (1/6)	Hemosiderophages Observed (1/6)
**Kidney**						
Color	Normal	Normal	Normal	Normal	Normal	Pale brown (1/6)
Size *	Normal	Normal	Normal	Normal	Normal	Normal
RW g/Kg (Ⴟ ± SEM)	681 ± 60	700 ± 100	683 ± 50	668 ± 28	717 ± 32	675 ± 71
Histopathology	Normal	Normal	Normal	Normal	Normal	Normal

RW: Relative weight; SEM: standard error of the mean; Ⴟ: mean; Negative: untreated, uninfected animals; hLIMOX-Control: not infected animals treated with higher doses of an essential oil fraction of *Lippia alba* carvone chemotype enriched in limonene (170 mg/kg/day) and with added caryophyllene oxide (Sigma-Aldrich) (70 mg/kg/day); Positive: untreated, infected animals; LIMOX: infected animals treated with an essential oil fraction of *Lippia alba* carvone chemotype enriched in limonene (68.9 mg/kg/day) and with added caryophyllene oxide (Sigma-Aldrich) (70 mg/kg/day); LIMOXBNZ: infected animals treated with LIMOX and benznidazole (7.9 mg/kg/day); BNZ: infected animals treated with benznidazole (100 mg/kg/day). Data are representative of six independent experiments and values are expressed in mean ± SEM. * Size defined as ratio between maximum diameter and maximum length.

## Data Availability

Data is contained within the article.
